# Magnon dark modes and gradient memory

**DOI:** 10.1038/ncomms9914

**Published:** 2015-11-16

**Authors:** Xufeng Zhang, Chang-Ling Zou, Na Zhu, Florian Marquardt, Liang Jiang, Hong X. Tang

**Affiliations:** 1 Department of Electrical Engineering, Yale University, New Haven, Connecticut 06511, USA; 2 Department of Applied Physics, Yale University, New Haven, Connecticut 06511, USA; 3 Key Lab of Quantum Information, University of Science and Technology of China, CAS, Hefei 230026, China; 4 Institute for Theoretical Physics II, University of Erlangen-Nuremberg, Staudtstrasse 7, 91058 Erlangen, Germany; 5 Max Planck Institute for the Science of Light, Günther-Scharowsky-Straße 1/Bau 24, 91058 Erlangen, Germany

## Abstract

Extensive efforts have been expended in developing hybrid quantum systems to overcome the short coherence time of superconducting circuits by introducing the naturally long-lived spin degree of freedom. Among all the possible materials, single-crystal yttrium iron garnet has shown up recently as a promising candidate for hybrid systems, and various highly coherent interactions, including strong and even ultrastrong coupling, have been demonstrated. One distinct advantage in these systems is that spins form well-defined magnon modes, which allows flexible and precise tuning. Here we demonstrate that by dissipation engineering, a non-Markovian interaction dynamics between the magnon and the microwave cavity photon can be achieved. Such a process enables us to build a magnon gradient memory to store information in the magnon dark modes, which decouple from the microwave cavity and thus preserve a long lifetime. Our findings provide a promising approach for developing long-lifetime, multimode quantum memories.

## Abstract (web summary)

Yttrium iron garnet is a ferrimagnetic insulator which demonstrates robust photon-spin coupling in hybrid microwave cavity systems. Here, the authors demonstrate a magnon gradient memory based on the dark modes of a strongly-coupled system of multiple yttrium iron garnet spheres.

## Graphical abstract

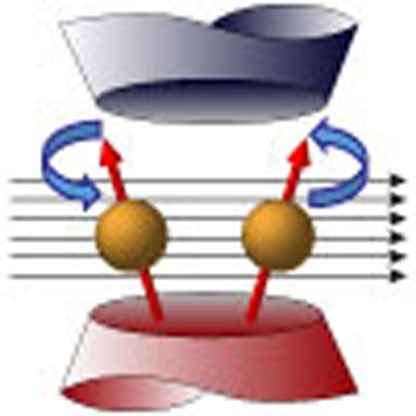

Hybrid systems provide a promising solution for coherent information storage by combining the long coherence time of spin ensembles with the power of superconducting circuits^1^,^2^,^3^. Researches utilizing ensembles ranging from cold atomic gases^4^, magnetic molecules^5^ and nuclear spin ensembles^6^ to rare-earth-ion-doped crystals^7^,^8^,^9^,^10^ and negatively charged nitrogen vacancy centres in diamond^11^,^12^,^13^,^14^,^15^,^16^ have been reported. Recently, people start to investigate the possibility of hybridizing yttrium iron garnet (YIG, Y_3_Fe_5_O_12_), a ferrimagnetic insulator, with microwave cavities. In the YIG crystal, the coherent photon-spin ensemble interaction is greatly enhanced by the high spin density and can even approach the ultrastrong coupling regime^17^,^18^,^19^,^20^,^21^,^22^. Compared with previous dilute spin ensemble systems, direct spin–spin interaction leads to collective magnon excitations that have a range of distinct advantages, such as low damping rate, uniform distribution and well-defined mode profiles and wave vectors. Particularly, each piece of YIG can be treated as a giant spin, with the flexibility to be individually manipulated in experiments.

In this work, we strongly couple multiple magnon modes with a microwave cavity resonance by placing multiple YIG spheres into a three dimensional cavity. With these coherently coupled magnons, collective effects^23^ such as a magnon dark mode (subradiant mode) that is decoupled from the environment, as well as enhanced interaction between a magnon bright mode (superradiant mode) and a microwave cavity mode are demonstrated. The spectrum of the magnons can be further adjusted by controlling the local magnetic field of each YIG sphere, which allows us to engineer the dissipation and tailor the dynamics of the microwave photon at will. With this method, we applied a magnetic field gradient, which induced the periodic evolution of the magnons between their temporal dark and bright modes, leading to a non-Markovian dynamics of the cavity energy that shows non-exponential decay and revival^24^. By optimizing the coupling condition^25^,^26^, good efficiency is obtained and our theory analysis indicates that a unity efficiency is achievable. Our experiment is performed at room temperature, demonstrating the coherent, long-lifetime, broadband and multimode gradient memory effect. A 100-ns storage time is achieved, which is limited by the magnon lifetime in our room temperature experiment and can possibly go up to several microseconds^27^ and even beyond^28^. Since the principle is entirely based on linear interactions, such a magnon memory can be readily scaled down to the quantum regime at millikelvin temperatures, providing a new approach besides the existing schemes for dilute spin ensembles^29^,^30^,^31^,^32^,^33^,^34^,^35^,^36^,^37^. This allows the realization of hybrid quantum memories that do not suffer from common problems such as inhomogeneous broadening.

## Results

### Magnon dark modes

**Figure 1:**
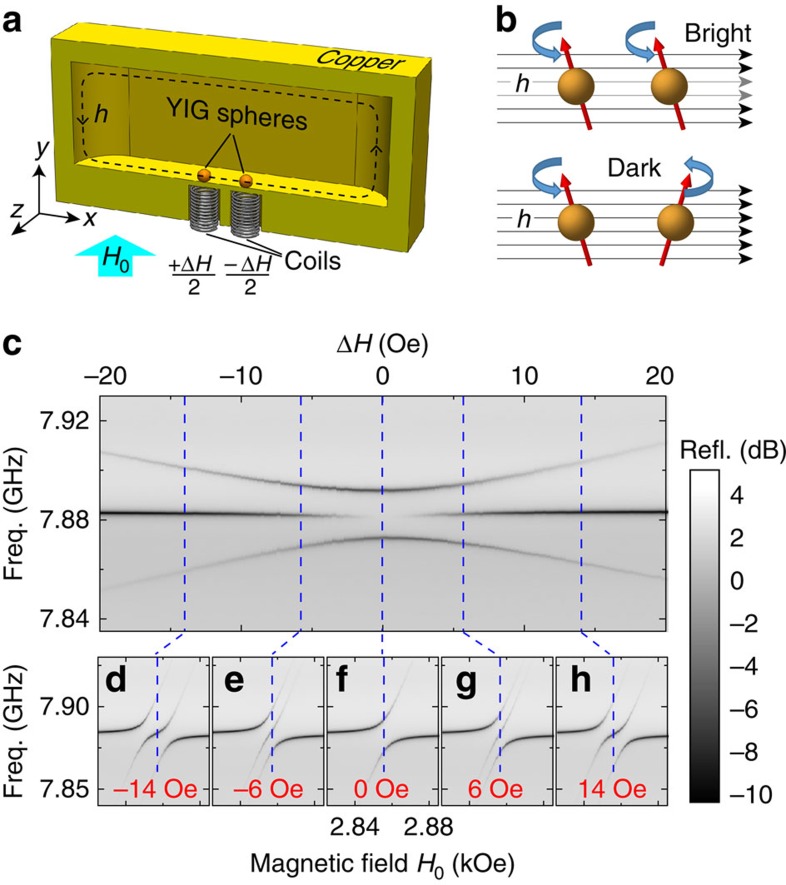
Magnon dark mode of two YIG spheres. (**a**) Device schematic including half of the copper cavity, two YIG spheres and two small coils. *H*_0_: external bias magnetic field; Δ*H*: magnetic field gradient generated by the small coils; *h*: magnetic field of the microwave resonance (TE_110_ mode) in the copper cavity. (**b**) Conceptual illustration of the magnon bright and dark modes: the bright mode is coupled to while the dark mode is isolated from the microwave magnetic field *h*. (**c**) Reflection spectra as a function of Δ*H* under an external bias magnetic field *H*_0_=2,858 Oe. (**d**–**h**) Reflection spectra as a function of bias magnetic field *H*_0_ at a fixed magnetic field gradient Δ*H*=−14, −6, 0, 6 and 14 Oe, respectively. Each dashed line in **c** corresponds to the dashed line in each of the spectral maps in **d**–**h**.

As a general situation, consider *N* identical YIG spheres loaded in a copper microwave cavity. The linear coupling between the uniform magnon modes in the YIG spheres and the cavity TE_110_ mode can be described by the Hamiltonian (Supplementary Note 1)


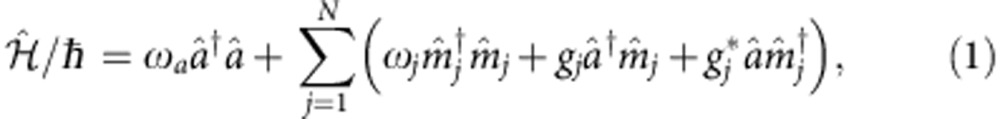


where 
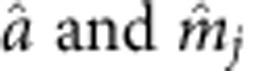
 are the bosonic operators associated with the photon and the uniform magnon modes, respectively, *g*_*j*_ is the coupling strength between the microwave mode and the magnon mode in the *j*-th YIG sphere with *j* ranging from 1 to *N* being the sphere index, *ω*_*a*_ is the resonance frequency of the TE_110_ mode of the copper cavity with the YIG spheres loaded but at zero bias field, *ω*_*j*_=*ω*_*a*_+[*j*−(*N*+1)/2]Δ*ω*_*m*_ are the evenly distributed magnon resonance frequencies and Δ*ω*_*m*_ is their frequency interval. Such a frequency distribution is obtained by biasing each YIG sphere at a magnetic field *H*_*j*_=*H*_0_+[*j*−(*N*+1)/2]Δ*H*, where *H*_0_=*ω*_*a*_/*γ* is the common external magnetic field that brings a YIG sphere on resonance with the microwave cavity TE_110_ mode, *γ*=2*π* × 2.8 MHz Oe^−1^ is the gyromagnetic ratio and Δ*H*=Δ*ω*_*m*_/*γ* is the magnetic field difference between neighbouring YIG spheres that is provided by the fine tuning of the small coils underneath each individual YIG sphere (Fig. 1a).

We first study the coherence of a simple system with two YIG spheres (*N*=2). As a result of the mode hybridization for non-zero *g*_1,2_, there exist three resonances in the cavity reflection spectrum when the two magnon modes are near resonance with the cavity TE_110_ mode but not on resonance, which agrees well with our experimental observation (Fig. 1c). By fixing Δ*H* and sweeping the external magnetic field around *H*_0_, two avoided crossings are observed in the reflection spectra (Fig. 1d), indicating the strong coupling between the cavity mode and the two magnon modes. When the frequencies of the two magnon modes (*ω*_1,2_) are brought closer by tuning Δ*H*, the absorption of the middle resonance becomes weaker, which eventually vanishes in the spectrum when the two magnon modes are brought simultaneously on resonance with the cavity mode (*ω*_1_=*ω*_2_=*ω*_*a*_) by turning off the gradient (Δ*H*=0), and such a transition is evident in Fig. 1c–h. The remaining two resonances emerge from the hybridization of the bright (superradiant) magnon mode with the cavity photon mode, while the resonance that has disappeared from the spectrum is the dark (subradiant) magnon mode, as it decouples from the microwave cavity (Supplementary Notes 2 and 3).

The concepts of the bright and dark modes are illustrated schematically in Fig. 1b. When the detuning field Δ*H*=0, the two magnon modes are on resonance *ω*_1_=*ω*_2_. Since, in addition, we have *g*_1_=*g*_2_, the bright mode is the hybridization of the two magnon modes that precess in phase 
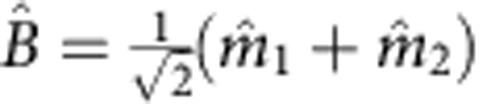
, while the dark mode is the hybridization of the two magnon modes that precess out of phase 
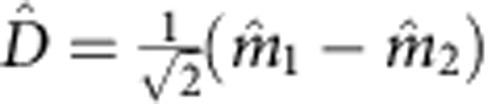
. For the bright mode, the coherent interactions between the magnons and photons are collectively enhanced, leading to an enhanced coupling strength 
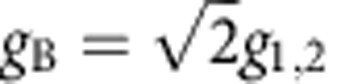
, which is verified in the avoided crossing spectrum where the splitting for Δ*H*=0 (2*g*=2*π* × 18.88 MHz, Fig. 1f) is 
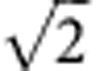
 times larger than that for Δ*H*=±14 Oe (2*g*=2*π* × 13.42 MHz, Fig. 1d,h). For the dark mode, the coupling of the two magnon modes with the cavity photons cancel each other, resulting in a vanishing coupling strength *g*_D_=0.

While the magnon bright mode can be used for information conversion with microwave photons, the dark mode is an ideal candidate for information storage because it decouples from the cavity and therefore has a very long lifetime^38^. A dark mode memory can be constructed if fast conversion between the bright and the dark modes is available, which in principle can be realized by rapidly tuning the magnetic bias field. However, the slow response of the local inductive coils prohibits the experimental realization of such an approach. Alternatively, we consider here a magnon gradient memory (MGM) using magnon temporal dark modes which eliminates the need of fast magnetic control. Such temporal dark modes are created by applying a magnetic field gradient (Δ*H*≠0) to uniformly detune the magnon modes around the cavity resonance, so unlike the steady bright and dark modes discussed above, they are not the eigenmodes of the Hamiltonian. Instead, the non-Markovian dynamics of such a coupled system leads to a temporally evolving interconversion between the temporal bright and dark modes, which provides a good solution for fast memory operations without adding extra controls.

For a device consisting of *N* YIG spheres, a frequency spectrum with *N*+1 resonances spaced at an interval of Δ*ω* (≈Δ*ω*_*m*_ for large *N*) can be obtained as a result of the coupling between the cavity mode and the uniformly distributed magnon modes. To achieve a memory with high efficiency, it is also important to ensure uniform coupling strengths for all the YIG spheres (*g*_*j*_=*g*_0_, see Supplementary Note 4 for details). The collective magnon modes from the *N* YIG spheres can be expressed as one temporal bright mode, expressed as


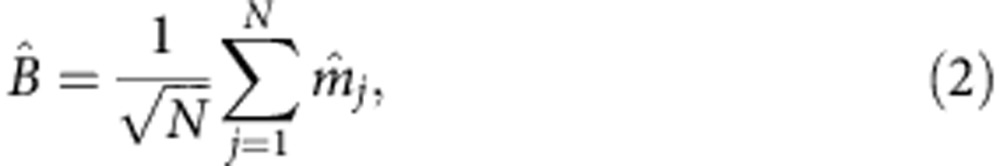


and *N*−1 temporal dark modes, with the *n*-th dark mode expressed as


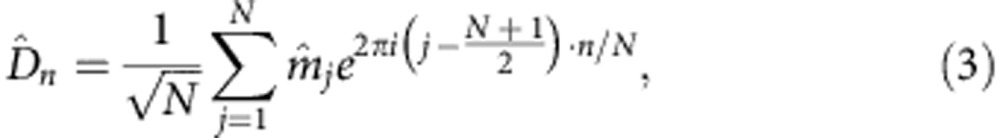


which does not couple to the cavity (Supplementary Note 3). Owing to the uniformly spaced detuning, the magnon modes described by equations (2) and (3) will convert from one to another with a time interval *T*/*N*, where *T*=2*π*/Δ*ω* is the evolution period of the collective magnon modes. During the storing process, the cavity photons are first converted to the temporal bright magnon mode, which is then successively converted to the temporal dark magnon modes that prevent the radiation loss. After one period, the system evolves back to the bright mode, and the information retrieves from the magnons and converts back to microwave photons. Both storing and retrieving processes are accelerated due to the superradiance effect. In such a configuration, no external control, such as refocusing pulse or gradient inversion, is required to retrieve the stored information. The performance of such an MGM depends on the number of YIG spheres. A larger *N* helps to improve the pulse reconstruction and suppress the off-peak ripples (Supplementary Note 4 and Supplementary Fig. 1).

### Magnon gradient memory

**Figure 2:**
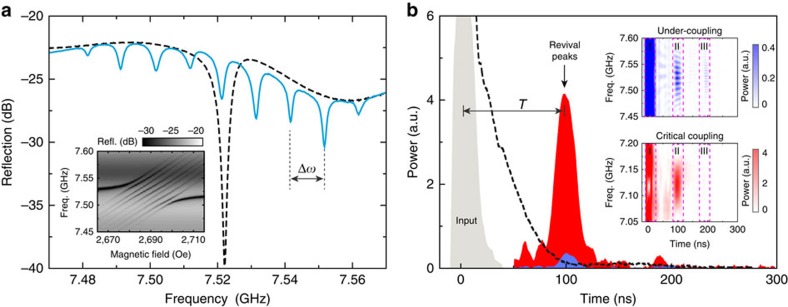
The magnon gradient memory. (**a**) Reflection spectra of the microwave cavity with eight YIG spheres at a bias field (*H*_0_) of 2,687 Oe (solid blue line) and 0 Oe (dashed black line), respectively. The frequency gradient of the magnon modes is tuned to be Δ*ω*=2*π* × 10 MHz. Inset: the reflection (Refl.) frequency (Freq.) spectra of the MGM at various bias magnetic fields. (**b**) The dynamics of the MGM output for a 15-ns pulsed microwave excitation (grey) at the under-coupling (blue) and critical-coupling (red) conditions with a frequency gradient of Δ*ω*=2*π* × 10 MHz. Dashed black curve shows the exponential decay for a critically coupled cavity resonance when the magnons are largely detuned. Operation condition for the blue curve: *ω*=2*π* × 7.522 GHz, *H*_0_=2,687 Oe; black: *ω*=2*π* × 7.522 GHz, *H*_0_=0 Oe; red: *ω*=2*π* × 7.120 GHz, *H*_0_=2,544 Oe. The input frequency for each case is adjusted accordingly with the cavity resonance shift induced by the change of the external coupling. Inset: pulse retrieval at various frequencies for the under-coupling and critical-coupling conditions, respectively. Zone I: instantaneous reflection for the input pulse; zone II: first revival peaks; zone III: second revival peaks.

In our experiments, we demonstrate such an MGM using eight YIG spheres (*N*=8, Supplementary Fig. 2). The large tunability of the magnon allows us to engineer the cavity dissipation with great flexibility and obtain a reflection spectrum (Supplementary Fig. 3) with a total of *N*+1=9 uniformly distributed hybrid modes, as shown in Fig. 2a (solid blue line), where Δ*ω*=2*π* × 10 MHz. Note that, in contrast to the stationary dark mode, the temporal dark modes can be detected in the spectrum since they convert back to the bright mode periodically. In other words, each of the nine resonance lines in the steady-state spectrum contains some component from the temporal bright mode, which can be detected. When a 15-ns-long pulsed microwave signal at a frequency *ω*=*ω*_*a*_=2*π* × 7.522 GHz is injected into the cavity with an external bias magnetic field of 2,687 Oe, it couples to the magnon bright mode and then converts to the magnon dark modes. The retrieval of the stored pulse takes place after a pre-programmed time *T*=2*π*/Δ*ω*=100 ns (blue peak in Fig. 2b), in sharp contrast with the exponential decay when the magnons are strongly detuned by turning off the external bias magnetic field *H*_0_ (dashed black line in Fig. 2b). While the MGM works without the requirement of any time-dependent control, expansions of the scheme would be possible when that control becomes available. For instance, on-demand recall can be achieved via dynamic control by rapidly turning off and on the magnetic field gradient. Additional control, such as gradient reversal, can be added to the control process. With the reversal process, the MGM works in a first-in-last-out mode similar as the gradient echo memory^33^,^39^,^40^,^41^ but with distinct differences from those dilute spin ensemble systems. While in gradient echo memory the reversal process is indispensable because of the inhomogeneous broadening in dilute spin ensembles, our MGM can work without the gradient reversal process, when the information is stored and retrieved in a first-in-first-out mode. Moreover, further dissipation engineering by controlling the field gradient would allow even more complicated manipulation of the output pulses, such as pulse shaping and pulse splitting. However, a fast magnetic field tuning at this time scale with reasonably large amplitude (around 50 Oe) is difficult to achieve and so it falls beyond the scope of this work and is left for future study.

The performance of the MGM can be significantly improved by optimizing the external coupling condition. Blue curves in Fig. 2a,b correspond to the under-coupling condition and therefore the retrieved pulse is very weak. A drastic boost of the revival pulse is obtained (red peak in Fig. 2b) by adjusting the coaxial probe to meet the critical-coupling (impedance matching) condition: *κ*_*a*,1_=*κ*_*a*,0_+*π*|*g*|^2^/Δ*ω*, where *κ*_*a*,0_ is the amplitude damping rate of the cavity resonance and *κ*_*a*,1_ is the coupling rate from the coaxial probe. The critical-coupling condition allows a complete energy conversion into the magnon bright mode (Supplementary Note 4 and Supplementary Fig. 4), and it also results in a maximized signal retrieval back to the microwave photon. Therefore, the first revival peak will contain all the stored information and there will be no successive revival peaks as observed in the under-coupling situation. Such analysis matches our measurement results shown in the insets of Fig. 2b: the second revival peaks (zone III) are relatively strong compared with the first revival peaks (zone II) for the under-coupling situation, while for the critical-coupling situation the second revival peaks disappear.

One advantage of the MGM is that its operation is not restricted to a narrow frequency range. A uniform frequency distribution exists in a wide frequency range as we vary the bias magnetic field (Fig. 2a, inset). Accordingly, broadband pulse retrieval process can be obtained in the time-domain measurement. Generally, the operation bandwidth can be determined by *N*Δ*ω*, and therefore, the delay-bandwidth product is set by *N*. In the experiment, we observed retrieval peaks in a broad frequency band of 80 MHz at a bias magnetic field of 2,687 Oe (Fig. 2b, insets), which is 1–2 orders of magnitude larger than the linewidth of the photon or magnon resonances. Note that when sweeping the input frequency, the revival peak intensity periodically varies for the under-coupling situation (Fig. 2b, inset, zone II), which can be attributed to the periodic variation in the coupling condition, while such a phenomenon disappears in the critically coupled situation thanks to the improved coupling condition.

**Figure 3:**
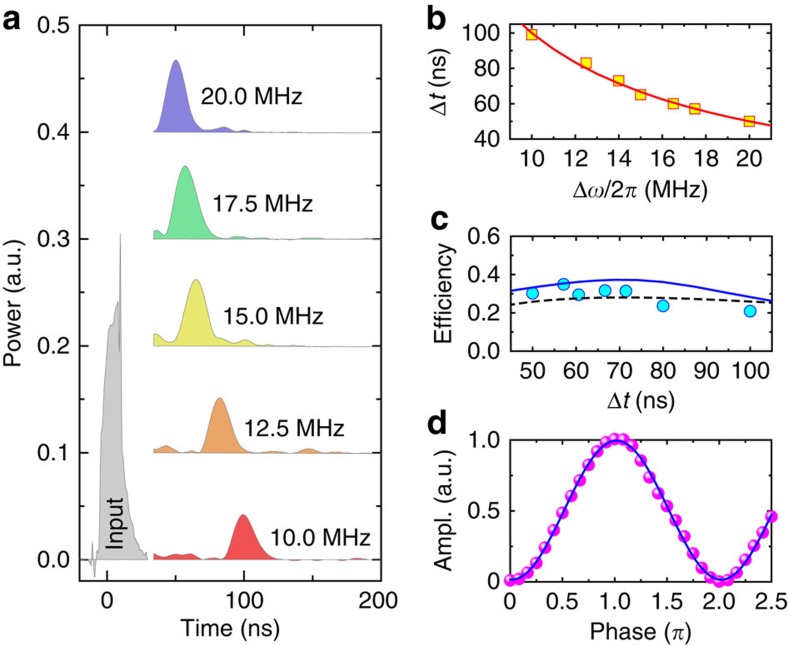
Characterization of magnon gradient memory. (**a**) Measured retrieval pulses for different frequency gradients Δ*ω*/2*π*. The input pulse is only shown for Δ*ω*/2*π*=10 MHz and remains the same for the other cases. (**b**) Extracted storage time as a function of frequency gradient. The solid line is calculated by *T*=2*π*/Δ*ω*. (**c**) Retrieval efficiency as a function of the storage time obtained from the measurement (circles), the numerical fitting (solid blue line) and the calculation using equation (4) (dashed black line), respectively. (**d**) Measured interference between the retrieved and reference signals as a function of their phase difference. The solid line is a sinusoidal fitting. Ampl., amplitude.

We quantitatively characterized the MGM by measuring the delay time and the retrieval efficiency as a function of Δ*ω*, and the result is plotted in Fig. 3a and Supplementary Fig. 5b. As Δ*ω* reduces, the revival time *T* increases, and the measurement results perfectly follow the relation *T*=2*π*/Δ*ω* (Fig. 3b). The MGM retrieval efficiency is defined as the ratio of the output pulse energy to the input pulse energy. It has been extracted as a function of delay time and plotted in Fig. 3c (blue circles), showing an efficiency of about 30% under the critical-coupling condition. The extracted efficiencies match the numerical simulation results obtained using the same parameters as in the measurement (solid blue line). On the other hand, the efficiency for a rectangular input pulse under critical-coupling condition can be approximated using the asymptotic expression (Supplementary Note 4 and Supplementary Fig. 6)


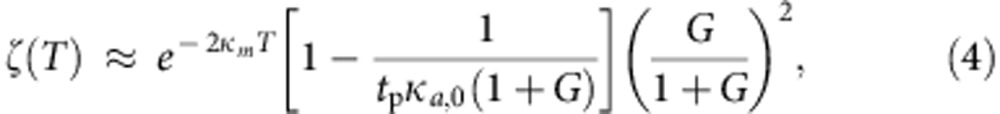


which is plotted in Fig. 3c (dashed black) for comparison. Equation (4) shows that the MGM efficiency depends on the figure of merit 
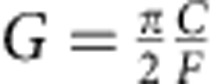
 for an input pulse with a duration of *t*_p_, where 
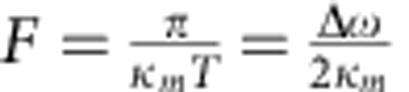
 is the finesse of the magnon gradient and 
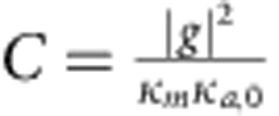
 is the magnon–photon interaction cooperativity.

The expression of the efficiency can be interpreted in three separate terms, which correspond to different physical processes. (i) The first term indicates the exponential decay of the stored energy with *T* due to the intrinsic magnon damping. This is the ultimate limitation of the memory storage time. (ii) The second term accounts for the spectrum overlap between the input microwave photon and the cavity-magnon system. Only input pulses with a bandwidth (∼2*π*/*t*_p_) smaller than the memory bandwidth (1+*G*)*κ*_*a*,0_ can be efficiently stored and retrieved. (iii) The last term represents the conversion efficiency between the temporal bright and dark modes, which can be calculated as 
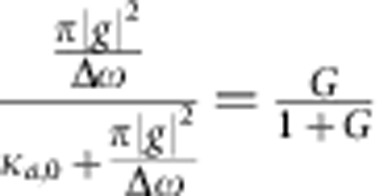
. Both the storage and retrieval processes are taken into account in equation (4). From the expression it is clear that by optimizing *C* and *F* we can further improve the MGM performance. In practice, *F* can be well controlled to be in the range of 10^1^−10^2^ and *C* in the order of 10^2^−10^4^, and thus *ζ*≈1 is achievable for real applications.

In addition to the high achievable efficiency, such an MGM preserves the signal coherence during the storage and retrieval process. Figure 3d plots the measured interference signal between the retrieved pulse (after a 100-ns storage time) and the input microwave signal. The periodic dependence of the interferometer output on the relative phase between these two signals indicates they are still phase coherent. A visibility as high as 96.9±1.5% can be extracted, proving the high coherence preservation feature of our MGM.

Another advantage of the MGM is its multimode operation capability, which is crucial for speeding up quantum computation protocols. The multimode nature of the MGM originates from the multiple degrees of freedom of the magnon modes. The MGM bandwidth *B* determines the minimum pulse length *t*_min_=2*π*/*B* and accordingly the maximum number of pulses that can be stored *n*_max_=*T*/*t*_min_. In ideal case with a large *N*, *B*=*κ*_*a*,0_+*π*|*g*|^2^/Δ*ω*, and therefore *n*_max_≈*π*|*g*|^2^/Δ*ω*^2^. For a limited number of magnon modes, *B*=*N*Δ*ω*, yielding a maximum pulse number *n*_max_=*N*, which equals the number of magnon modes, similar to the case in the atomic frequency comb (AFC) memories^42^.

**Figure 4:**
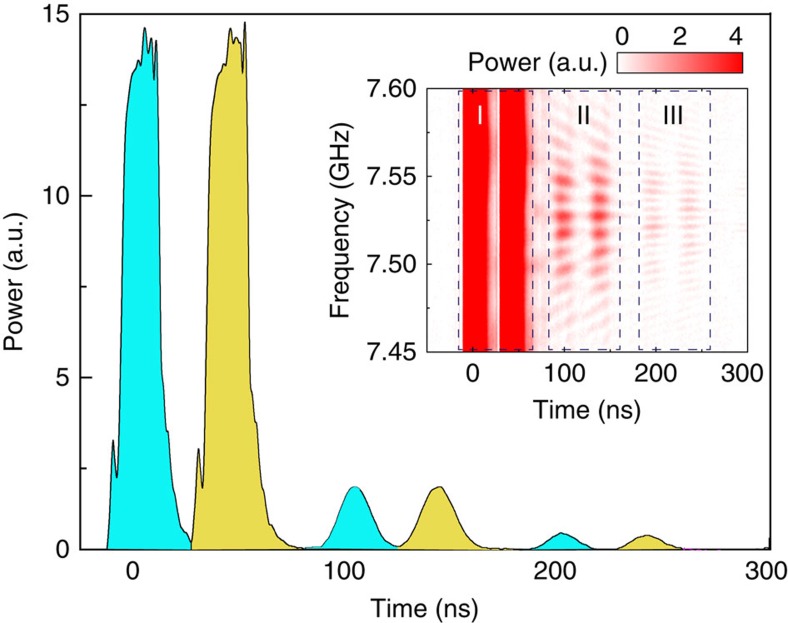
Multi-pulse storage in the MGM. Retrieved pulses for a double-pulse excitation. The two pulses are separated by 40 ns with a 15-ns duration each. Inset: double-pulse retrieval for various input frequencies at a bias field of 2,687 Oe.

Figure 4 plots the output of the MGM when two identical pulses are sent into the memory. Note that the MGM is under-coupled such that we can rely on the instantaneous reflection of the input pulse to control the pulse width and avoid overlap between the two pulses. In our linear system, the efficiency for the multimode operation remains the same as the single-pulse case. The two input pulses are 15 ns in duration and separated by 40 ns. Two pulses are retrieved from the memory after the pre-programmed storage time *T*=100 ns. As demonstrated in experiment (Fig. 4, inset), the multimode operation can also be achieved in a wide frequency range. The occasional pulse distortion at certain frequencies is a result of the non-ideality of the system such as the non-identical coupling strengths and decay rates of different YIG spheres, or the impact of the high-order magnon modes.

## Discussion

Although experimental imperfections such as non-uniform magnon frequencies and coupling strengths may affect the MGM performance (Supplementary Fig. 7), statistical analysis shows that our MGM is very robust against these imperfections (Supplementary Fig. 8). Moreover, the performance of our MGM can be further improved. For instance, the storage time can be extended by reducing the magnon damping through material purification to reduce the impurities, or through upgraded material fabrication technique that further reduces defects and surface roughness^43^,^44^. Moreover, it has been demonstrated in previous studies that there exist other types of magnon modes that have much narrower linewidths than the uniform mode in YIG spheres^28^, which can significantly increase the storage time if they can be adopted for MGM design.

Compared with previous memory schemes that are based on dilute spin ensembles^33^,^37^,^39^,^40^,^41^,^45^,^46^,^47^,^48^,^49^, our approach using a ferrimagnetic insulator has distinct advantages. As a result of the large spin density, the coupling strength between the magnon and the cavity photon is much stronger. Besides, the magnon in YIG forms well-defined modes due to the strong spin–spin interaction, and therefore they do not suffer from inhomogeneous broadening issues as in the dilute spin ensembles. Although high-order magnon modes can form in YIG, they did not affect our MGM performance since their frequencies are usually different from the fundamental magnon mode.

The storage and retrieval processes of our scheme can be related to the AFC scheme^37^,^42^,^45^,^46^,^47^. However, the AFC scheme only applies to dilute spin ensembles that have a broadened spectrum of optical transition frequencies, while our gradient memory scheme can be easily generalized to any system that is composed of harmonic oscillators. For instance, an array of mechanical oscillators is a promising candidate for opto- or electro-mechanical gradient memories, while a series of superconducting resonators with a SQUID as the tuning element can be used to build a superconductor gradient memory, which allows fast control and rich functionalities. Compared with other systems, our magnon system has its own unique advantages. For example, its dielectric construction has great compatibility with light, holding great potential for integrating microwave systems with photonic systems.

To summarize, we demonstrated the coherent coupling of multimode magnon resonances to a single microwave cavity. First we observed the magnon dark mode of two YIG spheres and then we expanded our scheme to the temporal magnon dark modes in a system consisting of eight YIG spheres. Owing to the non-Markovian dynamics of the system, it shows great potential in developing quantum memories that are broadband, multimode and do not require fast field switching. Our study shows a novel mechanism of manipulating magnons and microwave photons, paving the route towards magnon-based hybrid quantum memories, quantum repeaters and quantum networks^50^.

## Methods

### Sample preparation

The microwave cavities are machined from high-conductivity copper. The cavity housing two YIG spheres has a dimension of 43 × 21 × 9.6 mm^3^, with its TE_110_ resonating at 7.870 GHz, while the one housing eight YIG spheres has a dimension of 50 × 21 × 4.8 mm^3^, with its TE_110_ mode resonating at 7.522 GHz. All the YIG spheres are highly polished and have a diameter of 0.25 mm, with their 〈110〉 direction aligned parallel to the external bias magnetic field. The mounting ceramic rods that hold the YIG spheres are glued on the cavity wall, close to the maximum magnetic field of the cavity resonance to achieve strong magnon–photon coupling. Eight holes, 6.5 mm apart from each other, are drilled deep into (but not through) the cavity wall underneath the YIG spheres, where eight home-made small coils (200 turns, 6 mm in diameter, made of 32-gauge copper wires) are inserted with a 1-mm distance from the YIG spheres for fine tuning of the bias magnetic fields within a range of ±30 Oe (Fig. 1a and Supplementary Fig. 2a). Note that the positions of the YIG spheres are carefully adjusted as close to the small coils as possible so that the YIG spheres experience a uniform bias field, but not too close to the metal cavity wall, which disturbs the structural symmetry of the YIG spheres. These measures, combined with the fact that the cavity is considerably large compared with the YIG sphere, which provides very uniform microwave fields around the YIG spheres, ensure the preferential excitation of the uniform magnon modes while all the high-order modes get suppressed. The whole device assembly is inserted between the two poles of a water-cooled electromagnet, which provides the strong external bias magnetic field.

### Microwave characterization

The frequency spectra are taken using a vector network analyser by measuring the reflection signal from the coaxial probe, which accesses the cavity mode through a small hole in the cavity wall. The external coupling can be tuned by adjusting the position of the coaxial probe. In our measurements, the intrinsic losses of the cavity and magnon modes are *κ*_*a*,0_/2*π*=3 MHz and *κ*_*m*_/2*π*=0.72 MHz, respectively. The coupling strength with the cavity for each YIG sphere, *g*_*j*_/2*π* (*j*=1–8), varies between 8 and 12 MHz. The external coupling to the cavity is adjusted accordingly depending on the desired coupling condition. For example, *κ*_*a*,1_/2*π*=3 MHz for the under-coupling condition, while *κ*_*a*,1_/2*π*=34.4 MHz for the critical-coupling condition. The time traces are measured using a high-speed oscilloscope when the continuous-wave input microwave signal is modulated by a pulse generator through a transistor–transistor logic switch, which gives a rectangular pulse shape. To measure the coherence between the retrieved pulse and the input signal, the continuous-wave input signal is split into two branches before the pulse modulation, one of which, serving as the reference, is phase adjusted and combined with the retrieved signal before sending into an envelope detector. Then, the visibility of interference between the retrieved signal and the reference is measured by varying the reference phase. More details about the microwave characterization can be found in Supplementary Note 4.

### Theoretical derivation

In Supplementary Notes 1–4, details about the theory derivation and analysis of the dark mode and the gradient memory are provided.

## Additional information

**How to cite this article:** Zhang, X. *et al.* Magnon dark modes and gradient memory. *Nat. Commun.* 6:8914 doi: 10.1038/ncomms9914 (2015).

## Supplementary Material

Supplementary Information Supplementary Figures 1-8, Supplementary Notes 1- 4 and Supplementary References.
