# Supplementary material for: Magnon dark modes and gradient memory
Source: Nat Commun. 2015 Nov 16;6:8914. doi: 10.1038/ncomms9914 (PMC4660366; doi:10.1038/ncomms9914)
Supplement: Supplementary Information — Supplementary Figures 1-8, Supplementary Notes 1- 4 and Supplementary References. [file ncomms9914-s1.pdf]

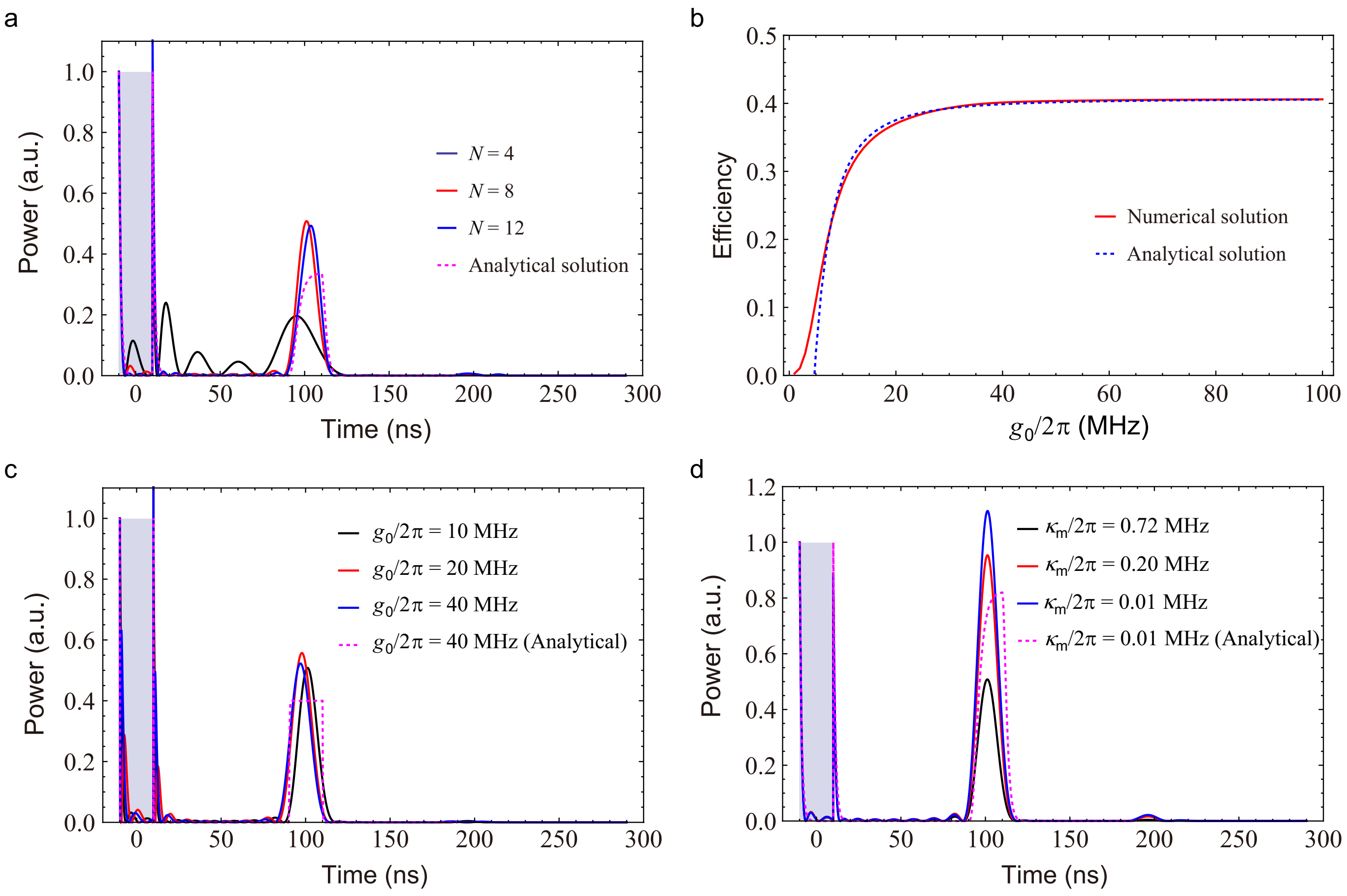

Supplementary Figure 1. **Calculated dependence of the MGM dynamics on different parameters. a,** Calculated dynamics of the MGMs with different YIG sphere numbers (N). **b,** Comparison of the numerical and analytical solutions at various coupling strengths. **c and d,** dynamics of the MGM with various coupling strengths and magnon dissipation rates, respectively. For each plot, the solid and dashed curves represent the numerical and analytical solutions, respectively. All parameters (beside the varying parameters) are taken from the experiments: $N = 8$, $g_0 = 2\pi \times 10$ MHz, $\Delta\omega = 2\pi \times 10$ MHz, $\kappa_{a,0} = 2\pi \times 3$ MHz, $\kappa_m = 2\pi \times 0.72$ MHz, $\kappa_{a,1} = \kappa_{a,0} + \frac{\pi|g_0|^2}{\Delta\omega}$ (critical coupling), and the duration of the rectangular input pulse (shaded areas in a, c, and d) is $t_{\rm p} = 20$ ns.

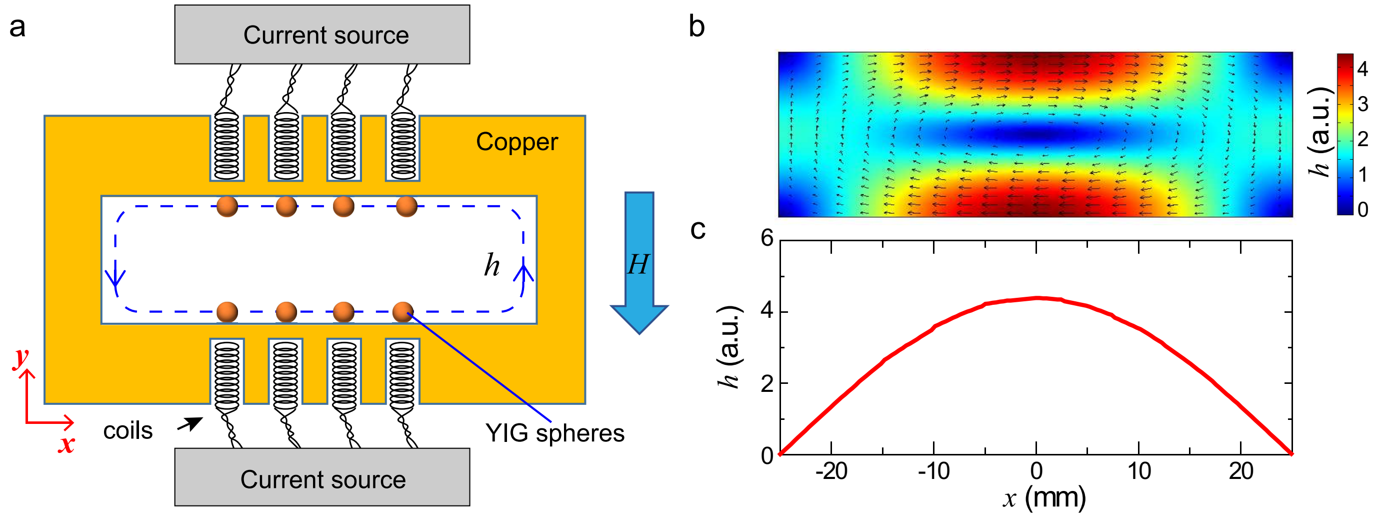

Supplementary Figure 2. **Design of the MGM device. a,** Device schematic of the MGM consisting of eight YIG spheres. **b,** The intensity and direction of magnetic field distribution of the cavity TE_{110} mode. Only the xy cross-section is shown because the magnetic field of this mode is almost uniform along the z direction. **c,** The cosine function-like magnetic field distribution along the x direction on the cavity wall where the YIG spheres are located.

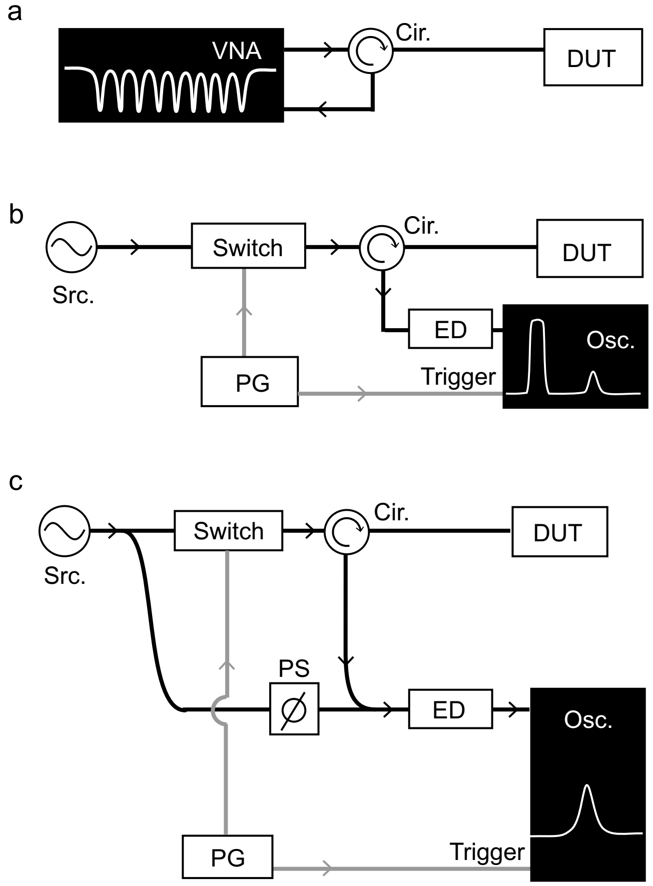

Supplementary Figure 3. **Schematics of the measurement setups. a,** The cavity reflection spectrum measurement setup. **b,** The time trace measurement setup. **c,** The measurement setup for the coherence of the retrieved pulse. VNA: vector network analyzer; DUT: device-under-test; Cir.: circulator; Src.: microwave source; ED: envelop detector; PG: pulse generator; Osc.: oscilloscope; PS: phase shifter.

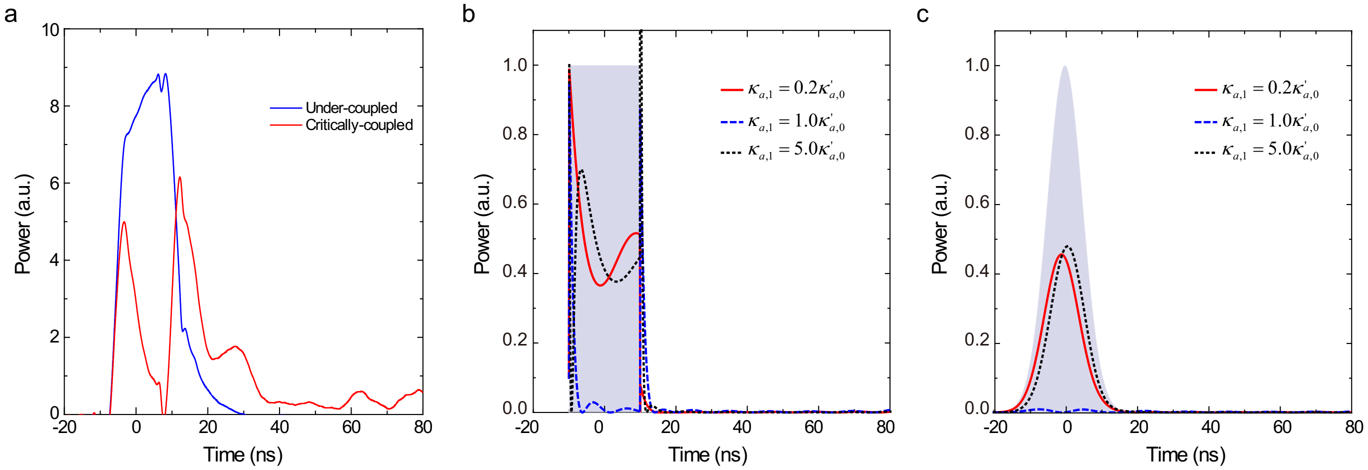

Supplementary Figure 4. **Input reflection at various coupling conditions.** **a,** Measured input reflections for microwave pulses to the microwave cavity with under-coupled ($\kappa_{a,1} < \kappa'_{a,0}$, blue curve) and critically coupled ($\kappa_{a,1} \approx \kappa'_{a,0}$, red curve) probes, respectively. **b,** The calculated cavity reflection for a rectangular pulse input (shaded area) at various coupling conditions. **c,** The calculated cavity reflection for a Gaussian input pulse (shaded area) using the same parameters as in b.

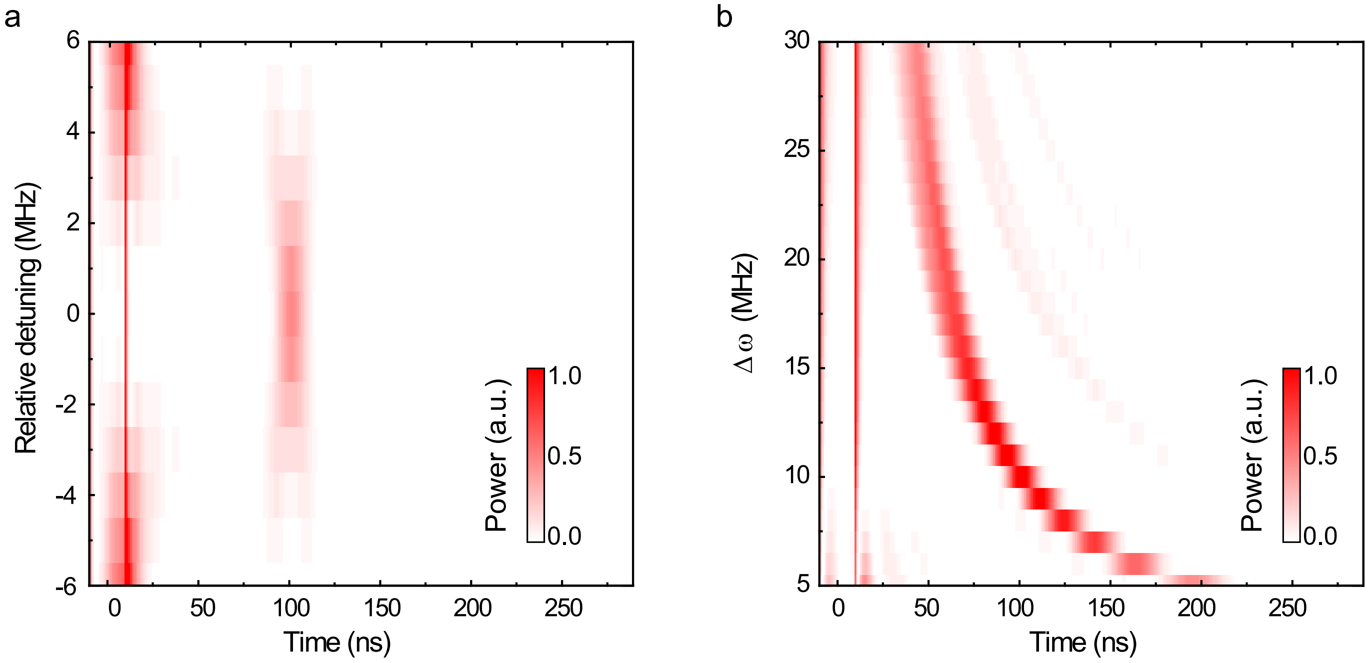

Supplementary Figure 5. **The calculated reflection signal dynamics at various conditions. a,** Calculated dynamics at various input microwave detunings $(\omega_{\text{in}} - \omega_a)/\Delta\omega$. **b,** Calculated dynamics at various magnon frequency gradients $\Delta\omega$. In the calculation, we assume all the magnon modes are identical, have uniform coupling strength, and are evenly distributed. Other parameters are from our experiment: $N = 8$, $g_0 = 2\pi \times 10$ MHz, $\Delta\omega = 2\pi \times 10$ MHz, $\kappa_{a,0} = 2\pi \times 3$ MHz, $\kappa_m = 2\pi \times 0.72$ MHz, $\kappa_{a,1} = \kappa_{a,0} + \frac{\pi |g_0|^2}{\Delta\omega}$ (critical coupling) and the input pulse duration is $t_{\text{p}} = 20$ ns.

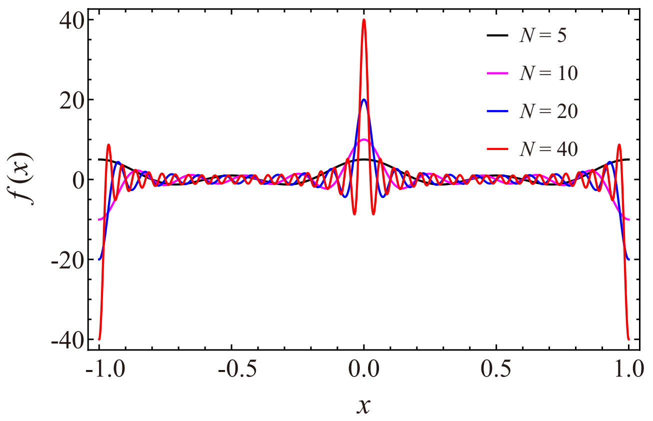

Supplementary Figure 6. The plot of the function $f(x) = \frac{\sin[\frac{N}{2}x]}{\sin[\frac{1}{2}x]}$.

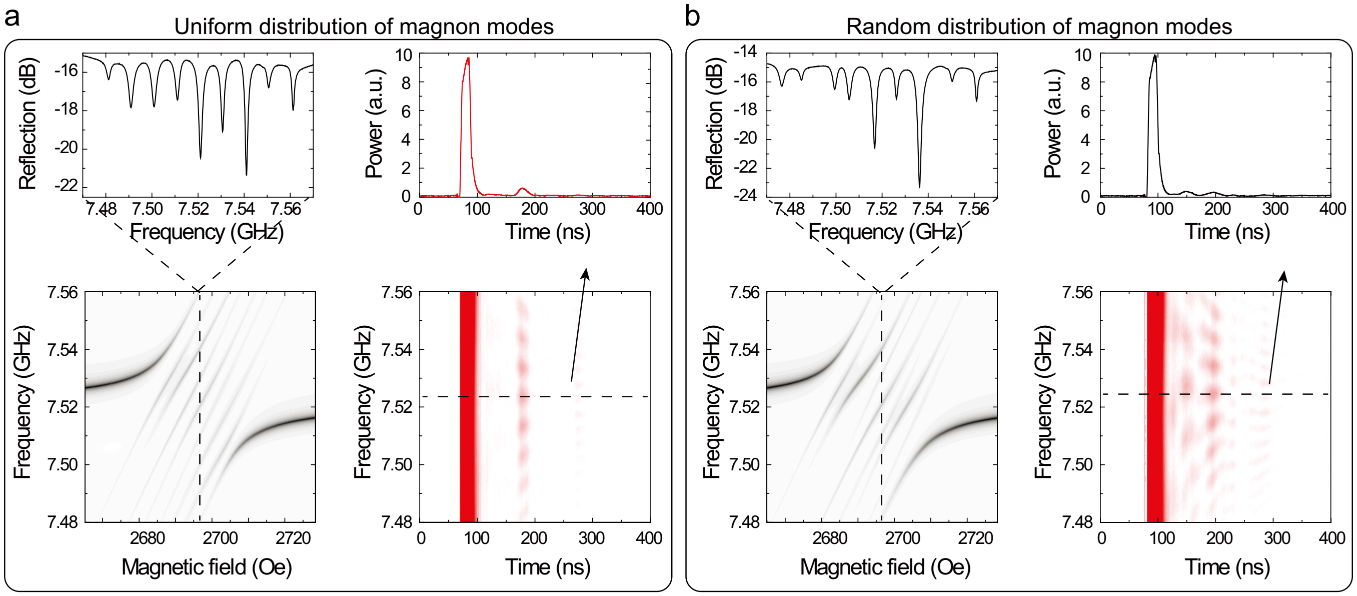

Supplementary Figure 7. **Comparison of the frequency spectrum and time trace for different magnon mode distributions.** **a,** The measurement results for uniformly distributed magnon modes. **b,** The measurement results for randomly distributed magnon modes.

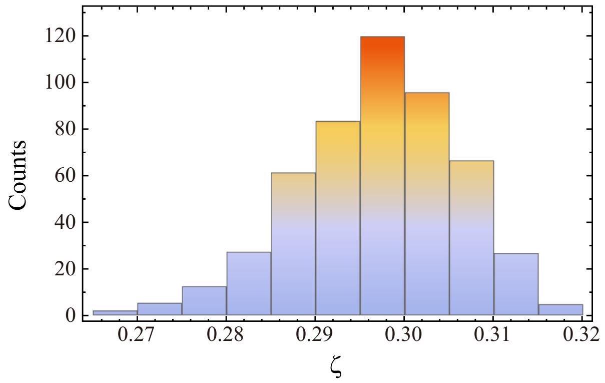

Supplementary Figure 8. **MGM efficiency statistics.** The statistical distribution of the numerically solved memory efficiency for randomly varied parameters g_j and ω_j.

Supplementary Note 1. Microwave photon-magnon strong coupling

The interaction between microwave photon and magnon (single mode) can be described by the Hamiltonian with the rotating-wave approximation

$$\hat{\mathcal{H}}/\hbar = \omega_a \hat{a}^\dagger \hat{a} + \omega_m \hat{m}^\dagger \hat{m} + g(\hat{a}^\dagger \hat{m} + \hat{a}\hat{m}^\dagger), \tag{1}$$

where $\hat{a}^\dagger$ ($\hat{a}$) is the creation (annihilation) operator for the microwave photon at frequency ω_a.For the uniform magnon mode, the collective spin excitations are approximately represented by the Boson operator $\hat{m}^\dagger(\hat{m})$ with Holstein–Primakoff approximation [1] and ω_m is the magnon frequency.

The coupling strength g between the two systems is [2]:

$$g = \frac{\eta}{2}\gamma\sqrt{\frac{\hbar\omega\mu_0}{V_a}}\sqrt{2Ns}, \tag{2}$$

where ω is the resonance frequency, γ is the gyromagnetic ratio, V_a is the modal volume of the microwave cavity resonance, μ_0 is the vacuum permeability, N is the total number of spins, and $s = \frac{5}{2}$ is the spin number of the ground state Fe^{3+} ion in YIG. The coefficient $\eta \leq 1$ describes the spatial overlap and polarization matching conditions between the microwave field and the magnon mode, which can be explicitly written as:

$$\eta^2 = \frac{(\overrightarrow{h}(\mathbf{r}) \cdot \overrightarrow{e_x})^2 + (\overrightarrow{h}(\mathbf{r}) \cdot \overrightarrow{e_y})^2}{\max\{|\overrightarrow{h}(\mathbf{r})|^2\}}, \tag{3}$$

where $\max\{|\overrightarrow{h}(\mathbf{r})|^2\}$ is the maximum magnetic field intensity of the cavity mode, and $\overrightarrow{h}(\mathbf{r})$ is the magnetic field amplitude at the location $(\mathbf{r})$ of the YIG sphere, $\overrightarrow{e_j}$ with $j = x, y, z$ are unit vectors and $\overrightarrow{e_z}$ is along the bias field direction.

Supplementary Note 2. Reflection spectrum

In our experiment, we measure the reflection spectrum under continues driving and the emission after pulsed excitations of the microwave cavity, which correspond to the eigenmode spectrum and the transient cavity output, respectively. In general, the equation of motion for the cavity photon is

$$\frac{d}{dt}\hat{a} = -i[\hat{a}, \hat{\mathcal{H}}] - i\sqrt{2\kappa_{a,1}}E_{\text{in}}(t)e^{-i\omega_l t}, \tag{4}$$

where $\hat{\mathcal{H}}$ is the system Hamiltonian, $\kappa_{a,1}$ is the cavity external coupling rate to the coaxial probe, $E_{\text{in}}(t)$ and ω_l are the amplitude and frequency of the input microwave.

In the rotating frame of ω_l, the microwave electric field collected by the detector reads

$$E_{\text{out}}(t) = -E_{\text{in}}(t) + i\sqrt{2\kappa_{a,1}}\hat{a}(t) \tag{5}$$

For a continuous stable input $E_{in}(t) = E_{in}$, we measure the stationary spectra with the normalized spectrum expressed as

$$r(\omega_l) = -1 + i\sqrt{2\kappa_{a,1}}\frac{\hat{a}(\omega_l)}{E_{\text{in}}}. \tag{6}$$

For the memory operation, the retrieval of the microwave pulse is measured after the input pulse is stored, and the detected intensity is

$$I(t) = 2\kappa_{a,1}|\langle\hat{a}(t)\rangle|^2. \tag{7}$$

The efficiency of the MGM, defined as the energy ratio of the retrieval pulse to the input pulse, can be expressed as

$$\zeta = \frac{\int I(t)dt}{\int I_{\text{in}}(t)dt}. \tag{8}$$

Supplementary Note 3. Magnon dark modes

In general, the Hamiltonian of multiple linearly coupled magnon modes and the cavity mode reads:

$$\hat{\mathcal{H}}/\hbar = \omega_a \hat{a}^\dagger \hat{a} + \sum_{j=1}^{N} (\omega_j \hat{m}_j^\dagger \hat{m}_j + g_j \hat{a}^\dagger \hat{m}_j + g_j^* \hat{a} \hat{m}_j^\dagger). \tag{9}$$

When deriving the equations of motion, both the magnon dissipation and the cavity dissipation (including the external coupling) are considered. In principle, the input-output theory shows that this gives a damping term as well as a fluctuation (including vacuum and thermal) term. To keep the notation simple, we will, from now on, use symbols like a and m_j to refer to the expectation values of the underlying operators. Therefore, the fluctuation terms will be dropped. This is sufficient to obtain the linear scattering behavior of the system and to discuss its eigenmodes. With this proviso, the equations of motion for the cavity mode and the magnon modes are:

$$\frac{d}{dt} a = (-i\omega_a - \kappa_a) a - i \sum_j g_j m_j, \tag{10}$$

$$\frac{d}{dt} m_j = (-i\omega_j - \kappa_j) m_j - i g_j^* a. \tag{11}$$

Here, κ_a and κ_j are the total decay rates of the cavity photon and the magnon modes, respectively, with κ_a including both the internal dissipation ($\kappa_{a,0}$) as well as the coupling to the coaxial probe ($\kappa_{a,1}$).

Two magnon modes

As described in the main text, we first study the case with two magnon modes. For simplicity, let the two magnon modes be on-resonance with the cavity mode ($\omega_1 = \omega_2 = \omega_m$). Then the dynamics of the cavity field is

$$\frac{d}{dt} a = (-i\omega_a - \kappa_a) a - i(g_1 m_1 + g_2 m_2). \tag{12}$$

The cavity mode couples to the collective mode $B = (g_1 m_1 + g_2 m_2)/\sqrt{|g_1|^2 + |g_2|^2}$ as

$$\frac{d}{dt} B = (-i\omega_m - \kappa_m) B - i\sqrt{|g_1|^2 + |g_2|^2}\, a. \tag{13}$$

And the orthogonal mode $D = (g_2^* m_1 - g_1^* m_2)/\sqrt{|g_1|^2 + |g_2|^2}$ satisfies

$$\frac{d}{dt} D = (-i\omega_m - \kappa_m) D - i(g_1^* g_2^* - g_2^* g_1^*) a/\sqrt{|g_1|^2 + |g_2|^2} \tag{14}$$

$$= (-i\omega_m - \kappa_m) D. \tag{15}$$

Therefore, D is completely isolated from the cavity mode, representing the dark mode that cannot be detected from the reflection spectrum of the microwave cavity.

Generalization to multiple magnon modes

For multiple magnon modes $N \geq 2$ and $\omega_j = \omega_1$ for $j = 2, \ldots, N$, the normalized bright mode is

$$B = \sum_{j=1}^{N} g_j m_j / \sqrt{\sum_{j=1}^{N} |g_j|^2}. \tag{16}$$

Then we have

$$\frac{d}{dt} a = (-i\omega_a - \kappa_a) a - i\sqrt{\sum_{j=1}^{N} |g_j|^2}\, B. \tag{17}$$

If the magnons are identical, the bright mode is a collective mode of all the magnon modes with the coupling strength being enhanced by a factor of

$$f = \sqrt{\sum_j |g_j|^2}/g_1 = \sqrt{N}. \tag{18}$$

There are $N-1$ other modes, orthogonal to the bright mode and decoupled from the cavity.

Temporal dark magnon mode

In the above analysis, all the magnon modes are on resonance with the cavity mode, and therefore the bright and dark modes are all the eigenmodes of the Hamiltonian. When the magnon modes are tuned off but close to resonance, situation would be different. As an example, we consider the case of two magnon modes. If the two magnon modes are detuned from the cavity mode as

$$\omega_1 = \omega_a + \Delta\omega/2, \tag{19}$$

$$\omega_2 = \omega_a - \Delta\omega/2, \tag{20}$$

then we can define the temporal bright mode at time t as (assuming $g_1 = g_2 = g_m$)

$$\widetilde{B}(t) = \frac{1}{\sqrt{2}} e^{-i\omega_a t}\left(m_1 e^{-i\Delta\omega t/2} + m_2 e^{i\Delta\omega t/2}\right). \tag{21}$$

At $t=0$, this mode is $B = \frac{1}{\sqrt{(2)}}(m_1+m_2)$, which is the same as static bright mode discussed above. But at $t = \frac{\pi}{\Delta\omega}$, the mode evolves to

$$\widetilde{B}(\frac{\pi}{\Delta\omega}) = \frac{-i}{\sqrt{2}} e^{-i\omega_a \pi/\Delta\omega}(m_1 - m_2) = -i e^{-i\omega_a\pi/\Delta\omega} D, \tag{22}$$

which is the dark mode that decouples from the cavity. Therefore, in this scenario, the temporal bright and dark magnon modes are not eigenmodes of the system and they are inter-convertible through time evolution.

Supplementary Note 4. Magnon gradient memory

Intuitive explanation

To realize a magnon memory device using the temporal magnon dark mode, we want: (1) the input photon can be converted to the collective magnon bright mode as quickly as possible, and therefore the photon energy will not dissipate too much due to the cavity intrinsic loss; (2) the bright mode can convert to the dark mode once the input photon is converted to the bright mode; (3) the retrieval of the photon is predictable and pre-programmable.

To fulfill these requirements, the system should consist of multiple magnon modes with equal detuning. Assuming the lifetime of the magnon and the cavity photon are $\tau_m = 1/2\kappa_m$ and $\tau_a = 1/2\kappa_a$, respectively, the retrieval period $T = \pi/\Delta\omega$, the conversion time from the bright mode to the dark modes $t_{\mathrm{B}\to\mathrm{D}} = T/N$ with N being the total number of the magnon modes, and the conversion time from the cavity photon to the magnon bright mode $t_{a\to\mathrm{B}} = \pi/\sqrt{N}g$, then we will have the following constraints

$$T \le \tau_m, \tag{23}$$

$$t_{\mathrm{B}\to\mathrm{D}} \ge t_{a\to\mathrm{B}}, \tag{24}$$

$$t_{a\to\mathrm{B}} \ll \tau_a, \tag{25}$$

which can be rewritten as

$$\Delta\omega \geq \frac{\pi}{\tau_m} = 2\pi \times \kappa_m, \tag{26}$$

$$g \geq \sqrt{N}\Delta\omega \geq 2\pi\sqrt{N} \times \kappa_m, \tag{27}$$

$$g \gg \frac{2\pi}{\sqrt{N}} \times \kappa_{a,0}. \tag{28}$$

A highly efficient magnon gradient memory requires the cooperativity $C = \frac{g^2}{\kappa_{a,0}\kappa_m} \gg 4\pi^2$. In our experiment, the hybrid YIG sphere–3D cavity structure can have a cooperativity C as large as 10^4, and therefore it is very promising for memory applications.

Dynamics

In an ideal magnon gradient memory, we have

$$\omega_j = \omega_a - \frac{N-1}{2}\Delta\omega + j\Delta\omega \tag{29}$$

for the uniform magnon mode in the $j-$th YIG sphere. Since

$$\frac{d}{dt}m_j = (-i\omega_j - \kappa_j)m_j - ig_j^* a, \tag{30}$$

we have the formal solution for the magnon time-evolution (where $m_j(0) = 0$):

$$m_j(t) = -ig_j^* \int_0^t a(\tau)e^{(-i\omega_j-\kappa_j)(t-\tau)}d\tau. \tag{31}$$

Then, the cavity dynamics is

$$\frac{d}{dt}a = (-i\omega_a - \kappa_a)a - \sum_j |g_j|^2 \int_0^t a(\tau)e^{(-i\omega_j-\kappa_j)(t-\tau)}d\tau \tag{32}$$

$$= (-i\omega_a - \kappa_a)a - \int_0^t a(\tau)\sum_j |g_j|^2 e^{(-i\omega_j-\kappa_j)(t-\tau)}d\tau. \tag{33}$$

For N identical YIG spheres with $g_j = g$ and $\kappa_j = \kappa_m$, we have

$$\begin{aligned}\sum_j |g_j|^2 e^{(-i\omega_j-\kappa_j)(t-\tau)} &= \sum_j |g_j|^2 e^{(-i\omega_a - i\frac{N-1}{2}\Delta\omega - ij\Delta\omega-\kappa_j)(t-\tau)} \\ &= |g|^2 e^{(-i\omega_a - i\frac{N-1}{2}\Delta\omega-\kappa_m)(t-\tau)}\sum_{j=1}^{N} e^{-ij\Delta\omega(t-\tau)} \\ &= |g|^2 e^{(-i\omega_a-\kappa_m)(t-\tau)}\frac{\sin[\frac{N\Delta\omega(t-\tau)}{2}]}{\sin[\frac{\Delta\omega(t-\tau)}{2}]}.\end{aligned} \tag{34}$$

Finally, the cavity dynamics is determined by the following equation

$$\frac{d}{dt}a(t) = (-i\omega_a - \kappa_a)a(t) - |g|^2 \int_0^t a(\tau)e^{(-i\omega_a-\kappa_m)(t-\tau)}\frac{\sin[\frac{N\Delta\omega(t-\tau)}{2}]}{\sin[\frac{\Delta\omega(t-\tau)}{2}]}d\tau. \tag{35}$$

Asymptotic solution

As shown by Supplementary Fig. 6, we can take the approximation

$$\frac{\sin[\frac{N\Delta\omega(t-\tau)}{2}]}{\sin[\frac{\Delta\omega(t-\tau)}{2}]} \approx 2\pi \sum_l (-1)^{l(N-1)} \delta[\Delta\omega(t-\tau-l\frac{2\pi}{\Delta\omega})] \tag{36}$$

for $N \gg 1$, where $\delta(x)$ is the Dirac delta function.

Therefore, we can describe the cavity dynamics for $N \gg 1$ as

$$\begin{aligned}\frac{d}{dt}a(t) &= (-i\omega_a - \kappa_a)a(t) - 2\pi |g|^2 \int_0^t a(\tau) e^{(-i\omega_a-\kappa_m)(t-\tau)} \sum_{l=-\infty}^{\infty} (-1)^{l(N-1)} \delta[\Delta\omega(t-\tau-l\frac{2\pi}{\Delta\omega})] d\tau \\ &= (-i\omega_a - \kappa_a)a(t) - \frac{\pi |g|^2}{\Delta\omega} a(t) - \sum_{l\neq 0} (-1)^{l(N-1)} \frac{2\pi |g|^2}{\Delta\omega} a(t - l\frac{2\pi}{\Delta\omega}) e^{(-i\omega_a-\kappa_m)(t-\tau)}. \end{aligned} \tag{37}$$

For $t < \frac{2\pi}{\Delta\omega}$, the cavity photon experiences single exponential decay

$$\frac{d}{dt}a(t) = (-i\omega_a - \kappa_a)a(t) - \frac{\pi |g|^2}{\Delta\omega} a(t), \tag{38}$$

with solution

$$a(t) = a(0) e^{-(i\omega_a + \kappa_a + \frac{\pi |g|^2}{\Delta\omega})t}. \tag{39}$$

We can see that the cavity photon decay is enhanced by $\frac{\pi |g|^2}{\Delta\omega}$.

For $\frac{2\pi}{\Delta\omega} \leq t \leq 2\frac{2\pi}{\Delta\omega}$, we have

$$\frac{d}{dt}a(t) = (-i\omega_a - \kappa_a - \frac{\pi |g|^2}{\Delta\omega})a(t) + \frac{2\pi |g|^2}{\Delta\omega} a(t - \frac{2\pi}{\Delta\omega}) e^{(-i\omega_a-\kappa_m)\frac{2\pi}{\Delta\omega}}, \tag{40}$$

$$= (-i\omega_a - \kappa_a - \frac{\pi |g|^2}{\Delta\omega})a(t) + \frac{2\pi |g|^2}{\Delta\omega} a(0) e^{(-i\omega_a-\kappa_m)\frac{2\pi}{\Delta\omega}} e^{-(i\omega_a+\kappa_a+\frac{\pi|g|^2}{\Delta\omega})(t-\frac{2\pi}{\Delta\omega})}. \tag{41}$$

Therefore, the cavity photons show a revival due to the energy of magnons coupling back to the cavity

$$a(t) = -\frac{2\pi |g|^2}{\Delta\omega} a(0) e^{(-i\omega_a-\kappa_m)\frac{2\pi}{\Delta\omega}} (t - \frac{2\pi}{\Delta\omega}) e^{-(i\omega_a+\kappa_a+\frac{\pi|g|^2}{\Delta\omega})(t-\frac{2\pi}{\Delta\omega})}. \tag{42}$$

Scattering picture

Above, we have adopted a direct solution of the temporal equations of motion. Alternatively, one can also discuss the MGM as a scattering problem in frequency space. We employ the equations of motion, Supplementary Eqs. (10) and (11), supplement them with the input field (as in Supplementary Eq. (4)), and obtain, in frequency space:

$$-i\omega a = (-i\omega_a - \kappa_a)a - i\sum_j g_j m_j - i\sqrt{2\kappa_{a,1}} E_{\rm in} \tag{43}$$

$$-i\omega m_j = (-i\omega_j - \kappa_m) m_j - i g_j^* a\,. \tag{44}$$

Here the functions a, m_j, and $E_{\rm in}$ are understood to be functions of the frequency ω.

These equations can be solved by eliminating m_j in favor of a and then applying the input-output relation Supplementary Eq. (5). This leads to the following result for the frequency-dependent reflection amplitude that gives $E_{\rm out}(\omega) = r(\omega) E_{\rm in}(\omega)$:

$$r(\omega) = \frac{[\omega_a - \omega + \Sigma(\omega)] + i(\kappa_{a,1} - \kappa_{a,0})}{-[\omega_a - \omega + \Sigma(\omega)] + i(\kappa_{a,1} + \kappa_{a,0})}\,. \tag{45}$$

At this point we have introduced the "MGM self-energy" that describes the collective effects of all the magnon modes acting on the cavity mode:

$$\Sigma(\omega) = \sum_j \frac{|g_j|^2}{\omega - \omega_j + i\kappa_m} \,. \tag{46}$$

The real part of Σ describes the effective frequency shift, while the imaginary part describes additional damping (induced by the magnon mode damping). The reflection amplitude can be decomposed into magnitude and phase shift:

$$r(\omega) = |r(\omega)| e^{i\theta(\omega)} \,. \tag{47}$$

In the ideal case without intrinsic losses ($\kappa_{a,0} = \kappa_m = 0$), we have $|r| = 1$. The time-delay of a scattered wave-packet is determined by the derivative of the phase shift with respect to frequency:

$$\tau(\omega) = \frac{\partial\theta(\omega)}{\partial\omega} \,. \tag{48}$$

The most important necessary condition for a useful memory is that this time-delay be constant over the bandwidth interval $N\Delta\omega$, i.e. the slope of θ should be constant. In the limit of small magnon-cavity coupling $g \to 0$ (and no intrinsic losses), each magnon resonance leads to a step of 2π in the phase shift θ. A finite g rounds off these steps. In the vicinity of each resonance, we can then approximate $\Sigma \approx g^2/(\omega - \omega_j)$, which leads to a phase shift rising like $\theta(\omega) - \theta(\omega_j) = 2\kappa_{a,1}(\omega - \omega_j)/g^2$. In order to have a constant overall slope of θ, we have to match this to the slope $T = 2\pi/\Delta\omega$ that is dictated by the spacing of resonances and which corresponds to the ideal storage time. That leads to the critical coupling condition (without intrinsic losses)

$$\frac{\pi g^2}{\Delta\omega} = \kappa_{a,1} \,. \tag{49}$$

We note that this condition remains true (in the present form) if intrinsic cavity losses are also incorporated, i.e. when $\kappa_{a,0} \neq 0$. The contribution from $\kappa_{a,0}$ cancels when deriving the condition. We note, however, that for finite $\kappa_{a,0}$ this condition slightly differs from the critical coupling condition derived below from demanding zero reflection of the input pulse. This is because, in general, for finite $\kappa_{a,0}$, the form of the critical coupling condition depends on the precise physical condition that is imposed. Still, one needs ideally $\kappa_{a,0} \ll \kappa_{a,1}$ to suppress unwanted losses and to avoid a resonance structure showing up in the magnitude $|r|$ of the reflection. In addition, the cavity mode acts like a filter, which should be broad enough to cover the whole magnon spectrum, i.e.

$$\kappa_{a,1} \gg N\Delta\omega \,. \tag{50}$$

Taking these two conditions together also implies $g \gg \kappa_{a,1}/\sqrt{N}$, as stated already in Supplementary Eq. (28). In addition, to keep the magnon losses small (ensuring $|r|$ close to 1), one needs $\kappa_m \ll \Delta\omega/2\pi$, as stated in Supplementary Eq. (26).

Critical coupling condition

From Supplementary Eq. (39), the microwave pulse input into the cavity couples to the external coupling channel (rate $\kappa_{a,1}$) and the temporal bright mode ($\frac{\pi|g_0|^2}{\Delta\omega}$), and also dissipates ($\kappa_{a,0}$) due to intrinsic radiation and absorption losses. Therefore, the full internal loss of the cavity is actually $\kappa'_{a,0} = \kappa_{a,0} + \frac{\pi|g_0|^2}{\Delta\omega}$. To suppress the reflection of the input pulse, the critical coupling (impedance matching) condition $\kappa_{a,1} = \kappa'_{a,0} = \kappa_{a,0} + \frac{\pi|g_0|^2}{\Delta\omega}$ is desired. The storage and retrieval efficiency of the MGM can be greatly affected by the critical coupling condition of the MGM with the coaxial probe. If the MGM is under-coupled, a large portion of the energy will be reflected as the signal gets in and out of the cavity, and as a result the MGM efficiency becomes low.

In our measurements, we did observe a strong reflection peak at the time of input for the undercoupling situation, and such reflection peak can be eliminated after adjusting the external coupling rate to meet the critical coupling condition, as plotted in Supplementary Fig. 4a. Such an observation agrees well with the theoretical predication (Supplementary Fig. 4b). Note that there is still some residual reflection for the critical coupling condition, which is attributed to the step response of the MGM to the rectangular input pulse (both the rising and falling edges). This imperfection can be suppressed by choosing Gaussian pulse inputs (Supplementary Fig. 4c).

Photon retrieval

From Supplementary Eq. (42), after one period $T = \frac{2\pi}{\Delta\omega}$ the stored energy will couple back to the cavity, which leads to the retrieval of the photons. We can solve for the detected microwave amplitude by the input-output formula Supplementary Eq. (5), with the input microwave also taken into account. Substituting the experiment parameters into the equation of the cavity field, with the input rectangular pulse duration $t_{\mathrm{p}} = 20$ ns, we obtained the dynamics in Supplementary Fig. 1a. The analytical and numerical results agree well with each other. With the given parameters, the numerical solution shows a memory efficiency of $\zeta = 0.33$, which is the integrated total output energy in the first retrieval peak as compared with that of the input pulse.

From the asymptotic solution, we can derive the efficiency of the magnon gradient memory as

$$\zeta \approx e^{-2\kappa_m \frac{2\pi}{\Delta\omega}} \left[1 - \frac{1}{t_{\mathrm{p}}\left(\kappa_{a,0} + \frac{\pi|g|^2}{\Delta\omega}\right)}\right] \left(\frac{\frac{\pi|g|^2}{\Delta\omega}}{\kappa_{a,0} + \frac{\pi|g|^2}{\Delta\omega}}\right)^2 \tag{51}$$

for a rectangular input pulse under critical coupling condition (which demands zero reflection). Note that the pulse duration is limited by the external coupling rate: $t_{\mathrm{p}} > 1/\kappa_{a,1} = 1/(\kappa_{a,0} + \frac{\pi|g|^2}{\Delta\omega})$.

In Supplementary Fig. 1b, we plot the efficiency as a function of the coupling strength g_0. A comparison of the numerical results with the asymptotic solutions shows that they agree well with each other for $g/2\pi \geq 7$ MHz. The efficiency saturates to $\zeta = e^{-2\kappa_m \frac{2\pi}{\Delta\omega}} \approx 0.403$ for $\frac{\pi|g_0|^2}{\Delta\omega} \gg \kappa_{a,0}$, which is limited by the intrinsic loss of the magnon. Supplementary Fig. 1c plots the behavior of the memory for various coupling strengths, which shows the saturated retrieval peak at 100 ns. If we can reduce the magnon dissipation rate, as shown in Supplementary Fig. 1d, the retrieval efficiency approaches unity. If operating at low temperatures, the magnon linewidth can be reduced to 0.042 MHz [3], which will lead to a saturated efficiency of 0.95 for a storage time $\frac{2\pi}{\Delta\omega} = 100$ ns.

By numerical calculation, we further studied the memory for different input pulse detuning and frequency gradient of the magnons. From our analytical solutions, we can expect the effective bandwidth of the MGM to be about $\kappa'_{a,0} = \kappa_{a,0} + \frac{\pi|g_0|^2}{\Delta\omega} \approx \frac{\pi|g_0|^2}{\Delta\omega}$ for $N \to \infty$. However, in experiments we have a finite N, and therefore the bandwidth is also limited by the bandwidth of the gradient magnon spectrum, which is about $N\Delta\omega$. Therefore, the highly efficient memory works in a bandwidth of $\min\{\frac{\pi|g_0|^2}{\Delta\omega},\ N\Delta\omega\}$. From Supplementary Fig. 5a, the bandwidth is about $8\Delta\omega$, which is consistent with our theory. Supplementary Fig. 5b shows that the storage time is inversely proportional to $\Delta\omega$, which also agrees with our expectation.

Measurement scheme

Supplementary Fig. 2a illustrates the device schematic of the MGM with eight YIG spheres. Note that the radius (3 mm) of the small coils is much larger than the YIG sphere radius to ensure the magnetic fields generated by the coils are uniform at the position of the YIG sphere (1 mm above the coil). As a result, the coils have to be placed 6.5 mm apart from one another, and so are the YIG spheres. Since the magnetic field of the cavity TE_{110} mode has a cosine distribution along the $\vec{x}$ direction inside the cavity (Supplementary Figs. 2b & c), different YIG spheres experience different magnetic field strengths. Therefore the coupling strength of the magnon modes with the cavity mode are not identical, and this contributes to the non-ideality of the MGM. Such non-ideality is unavoidable, but in our experiment efforts such as placing the YIG spheres as close to the center as possible have been taken to reduce the non-ideality in the device.

There are three types of measurement involved in our experiments.

(1) First is the cavity reflection spectrum measurement. The input signal is provided and the reflected signal is detected by a vector network analyzer, as indicated in Supplementary Fig. 3a. A circulator is used to separate the input and reflected signals to avoid undesired interference.

(2) The second type of measurement is the time trace measurement, which is carried out using a high-speed oscilloscope (Supplementary Fig. 3b). The signal from a microwave source is modulated by a pulse generator through a transistor–transistor logic (TTL) switch to obtain a pulsed microwave signal, which is sent into the device, and then a retrieval pulse can be measured after the pre-programmed retrieval time.

(3) The third type of measurement is the coherence measurement, as shown in Supplementary Fig. 3c. The scheme is similar to the time trace measurement but with an interferometer added. The input signal is split into two branches, one of which is used as the reference to interfere with the output signal. The setup is very similar to the Mach-Zehnder interferometer commonly used in optical measurements. By varying the phase of the reference, the amplitude of the interference signal changes, and their relation is measured to characterize the retrieval pulse coherence.

Experimental imperfections

The operation of the MGM requires the hybrid magnon-photon modes evenly distributed in the frequency domain, which will give a perfect constructive interference at the pre-programmed retrieval time T, and best suppression of the fringes at other times. Imperfection in the frequency distribution will result in deteriorated signal re-construction. This is clearly illustrated by the comparison given in Supplementary Fig. 7. For a uniformly distributed spectrum, the retrieval pulse is very clean; while for the non-uniformly distributed spectrum, the retrieval pulse is severely distorted.

In our experiments, we can precisely tune the small coil to obtain a near perfect gradient for the magnon modes. The small experimental imperfections only have very slight effects on the performance of the MGM. To investigate the influence of the experimental imperfection on the efficiency of the MGM, we simulated the efficiencies for various parameters, with random perturbations to the ideal case, where

$$g_j = g_0 \times (1 + \xi_j), \tag{52}$$

$$\omega_j = \omega_a + (j - \frac{N-1}{2})\Delta\omega_m + \Delta\omega_m \times \xi_j. \tag{53}$$

Here, $\xi_j \in [-0.1, 0.1]$ is a uniformly distributed random variable, which means the variations of magnon frequency and coupling strengths are within a range of $\pm 10\%$.

The results for 500 sets of different parameters are shown in Supplementary Fig. 8, with the mean value of ζ being about 0.30, while the standard deviation is 0.009. For such a high imperfection up to 10%, the MGM still shows a very good ability for signal re-construction. Therefore, the performance of the MGM is very robust against experimental imperfections.

Supplementary References

[1] Holstein, T. & Primakoff, H. Field dependence of the intrinsic domain magnetization of a ferromagnet. *Phys. Rev.* **58**, 1098–1113 (1940).

[2] Zhang, X., Zou, C.-L., Jiang, L. & Tang, H. X. Strongly coupled magnons and cavity microwave photons. *Phys. Rev. Lett.* **113**, 156401 (2014).

[3] Spencer, E. G., LeCraw, R. C., & Linares, R. C. Jr. Low-temperature ferromagnetic relaxation in yttrium iron garnet. *Phys. Rev.* **123**, 1937–1938 (1961).
